# Evidence-practice gap analysis in the role of tick in brucellosis transmission: a scoping review

**DOI:** 10.1186/s40249-023-01170-4

**Published:** 2024-01-08

**Authors:** Rui Ma, Chunfu Li, Ai Gao, Na Jiang, Xinyu Feng, Jian Li, Wei Hu

**Affiliations:** 1 School of Life Sciences, Inner Mongolia University, Hohhot, 010070 China; 2 School of Global Health, Chinese Center for Tropical Diseases Research, Shanghai Jiao Tong University School of Medicine, Shanghai, 20025 China; 3 One Health Center, Shanghai Jiao Tong University-The University of Edinburgh, Shanghai, 20025 China; 4 Basic Medical College, Guangxi University of Chinese Medical, Nanning, 530005 Guangxi China; 5 Department of Infectious Diseases, Huashan Hospital, State Key Laboratory of Genetic Engineering, Ministry of Education Key Laboratory for Biodiversity Science and Ecological Engineering, Ministry of Education Key Laboratory of Contemporary Anthropology, School of Life Sciences, Fudan University, Shanghai, 200438 China

**Keywords:** Tick, *Brucella*, Detection, Human infection, Risk evaluation

## Abstract

**Background:**

Brucellosis is a zoonotic affliction instigated by bacteria belonging to the genus *Brucella* and is characterized by a diverse range of pervasiveness, multiple transmission routes, and serious hazards. It is imperative to amalgamate the current knowledge and identify gaps pertaining to the role of ticks in brucellosis transmission.

**Methods:**

We systematically searched China National Knowledge Infrastructure (CNKI), WanFang, Google Scholar, and PubMed on the topic published until April 23, 2022. The procedure was performed in accordance with the Systematic Reviews and Meta-Analyses extension for Scoping Reviews (PRISMA-ScR) guidelines. The selected articles were categorized across three major topic areas, and the potential data was extracted to describe evidence-practice gaps by two reviewers.

**Results:**

The search identified 83 eligible studies for the final analyses. The results highlighted the potential capacity of ticks in brucellosis transmission as evidenced by the detection of *Brucella* in 16 different tick species. The pooled overall prevalence of *Brucella* in ticks was 33.87% (range: 0.00–87.80%). The review also revealed the capability of *Brucella* to circulate in parasitic ticks' different developmental stages, thus posing a potential threat to animal and human health. Empirical evidence from in vitro rodent infection experiments has revealed that ticks possess the capability to transmit *Brucella* to uninfected animals (range: 45.00–80.00%). Moreover, significant epidemiological associations have been found between the occurrence of brucellosis in animals and tick control in rangelands, which further suggests that ticks may serve as potential vectors for brucellosis transmission in ruminants. Notably, a mere three cases of human brucellosis resulting from potential tick bites were identified in search of global clinical case reports from 1963 to 2019.

**Conclusions:**

It is imperative to improve the techniques used to identify *Brucella* in ticks, particularly by developing a novel, efficient, precise approach that can be applied in a field setting. Furthermore, due to the lack of adequate evidence of tick-borne brucellosis, it is essential to integrate various disciplines, including experimental animal science, epidemiology, molecular genetics, and others, to better understand the efficacy of tick-borne brucellosis. By amalgamating multiple disciplines, we can enhance our comprehension and proficiency in tackling tick-borne brucellosis.

**Supplementary Information:**

The online version contains supplementary material available at 10.1186/s40249-023-01170-4.

## Background

Brucellosis, a zoonotic disease caused by bacteria belonging to the genus *Brucella*, is a widespread affliction with a global presence in over 170 countries across all five continents [1]. The genus *Brucella* is composed of 12 species, including *B. abortus*, *B. ovis*, *B. melitensi*, *B. suis*, *B. canis*, *B. neotomae*, *B. ceti*, *B. pinripedialis*, *B. mieroti*, *B. vulpis*, *B. inopinata*, and *B. papionis* [2]. *B. melitensi* is the most significant pathogen responsible for human brucellosis worldwide, followed by *B. abortus* and *B. suis* [3]. Although Europe, Australia, and Canada have successfully eradicated brucellosis, brucellosis continues to be a significant concern in highly endemic areas such as Africa, parts of Asia, and Latin America [4]. Like other zoonotic diseases, brucellosis causes significant economic losses to animal husbandry due to decreased fertility, abortions, and lowered milk production. This poses a significant threat to the livestock, meat, and dairy industries [5]. Human health is also at risk, as brucellosis infections may result in long-term clinical symptoms, including sweating, joint pain, and fatigue [6]. Moreover, brucellosis profoundly impacts human social development, particularly among impoverished populations, impeding the creation of a healthy society and the sustainable development of human societies [7].

**Fig. 1:**
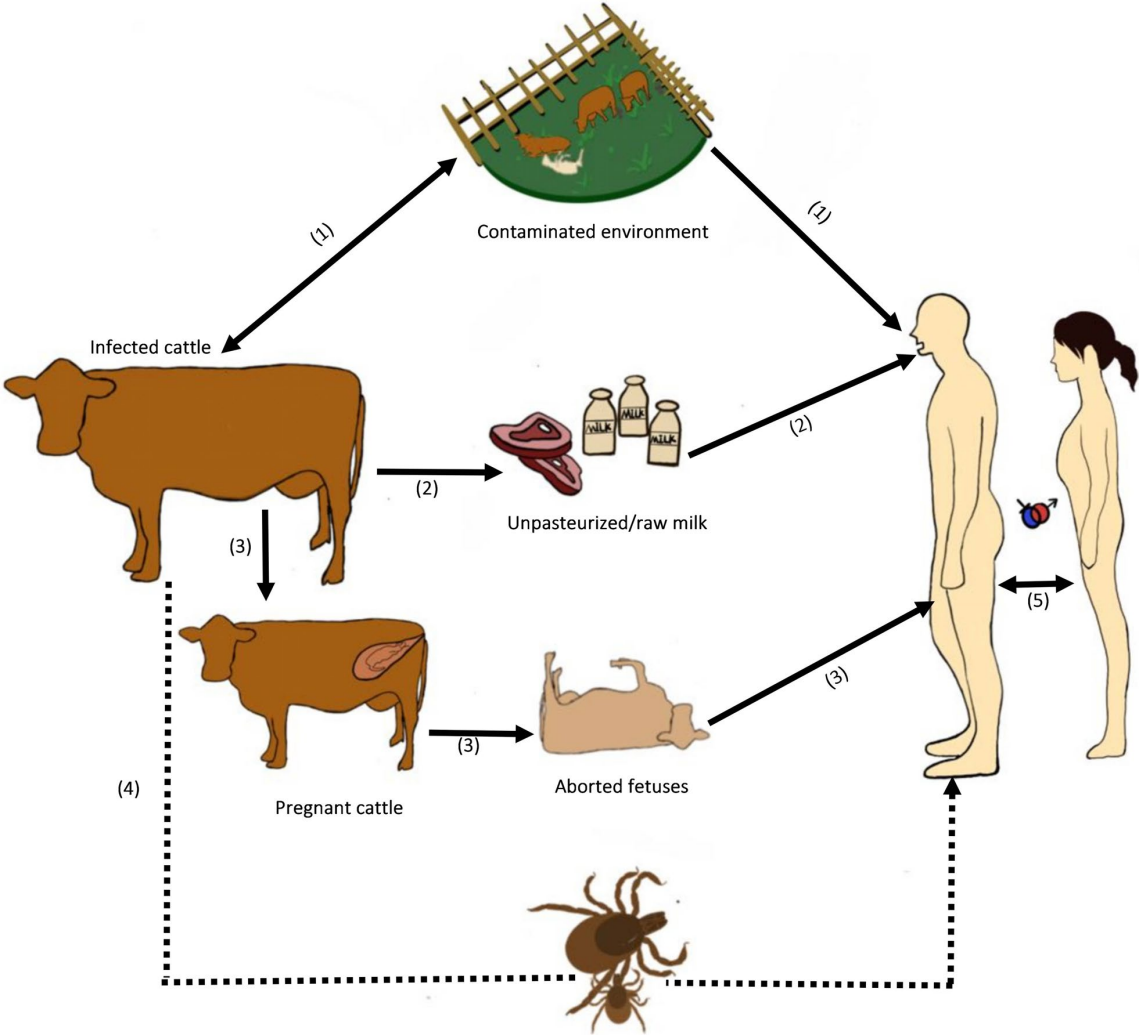
Common routes of transmission of brucellosis. (**1**) Respiratory transmission: respiratory inhalation of aerosols from *Brucella* contamination of the environment. (**2**) Gastrointestinal transmission: ingestion of raw unpasteurized dairy products and undercooked meat. (**3**) Contact transmission: contact with body fluids of infected animals. (**4**) Biological transmission: bite from infected ticks and other arthropods. (**5**) Sexual transmission. Note: the solid lines on the diagram denote confirmed transmission routes, while the dotted lines indicate potential transmission routes

The prevention and control of brucellosis is an intricate undertaking due to its diverse transmission routes, including respiratory, gastrointestinal, contact, biological, and sexual transmission (Fig. 1). Moreover, the disease's high susceptibility to relapse poses an additional challenge to managing and containing it. Therefore, it is crucial to implement robust measures to curb the spread of brucellosis and minimize the risk of relapse [8]. The prevention of brucellosis spread among farmers is often hindered by their insufficient knowledge and resources in effectively controlling outbreaks. Such limitations are primarily attributed to the inadequate regulatory procedures and compensation mechanisms implemented by regional administrations, coupled with the government’s inattention to monitoring and addressing the disease’s potential spread [9].

Although vaccines have been essential in preventing brucellosis, it's crucial to acknowledge their potential downsides. Toxic residues found in vaccines can be harmful to animals and humans, and their use can interfere with routine disease detection tests [10, 11]. It is noteworthy that brucellosis can present with a range of diverse symptoms, posing a diagnostic challenge for healthcare professionals. Consequently, misdiagnosis and delayed treatment are prevalent, ultimately jeopardizing patient safety by increasing the likelihood of complications [12]. Additionally, despite the annual registration of 500,000 cases of brucellosis, the World Health Organization (WHO) estimates that around a quarter of patients still go unreported and unrecorded. Consequently, the prevention and control of brucellosis continue to pose significant challenges [13, 14].

Tick-borne diseases (TBDs) are of great concern due to the potential for ticks to harbor a wide range of diseases that can be transmitted to humans, livestock, and wildlife [15, 16]. In recent years, changes in both microclimatic and macroclimatic conditions, as well as human behavior, have resulted in an expansion of the potential suitability areas, further increasing the likelihood of human exposure to these vectors [17]. This scoping review attempts to provide a deeper insight into the role of ticks in the transmission of brucellosis and identify gaps in the existing literature. To achieve this, we analyze the prevalence of *Brucella* in ticks, the detection of brucellosis in ticks, and the potential risk of tick-borne transmission. This thorough analysis will provide a more profound understanding of the relationship between ticks and brucellosis, and lay the groundwork for future research in this area.

## Methods

### Search strategy

The Systematic Reviews and Meta-Analyses extension for Scoping Reviews (the PRISMA-ScR) guidelines for conducting a scoping review were followed [18]. A literature search was conducted for publications up to April 2022. The review was undertaken to inform the role of ticks in brucellosis transmission. We listed all keywords related to the six key concepts of our research question: tick, *Brucella*, the prevalence of brucellosis in ticks, the detection methods of brucellosis, and the risk of tick-borne brucellosis. We used the search strategies presented in Additional file 1 to search the databases. We identified published studies based on combinations of our keywords in four bibliographic databases (two in Chinese and two in English): China National Knowledge Infrastructure (CNKI, https://www.cnki.net/), WanFang (www.wanfangdata.com.cn), Google Scholar (https://scholar.google.com), and PubMed (https://pubmed.ncbi.nlm.nih.gov). In conducting our research, we thoroughly examined the reference lists of all relevant studies in order to identify any additional research that may not have been initially detected through our electronic database searches. We did not try to obtain any unpublished studies, and there were no limitations on language.

### Inclusion and exclusion criteria

**Fig. 2:**
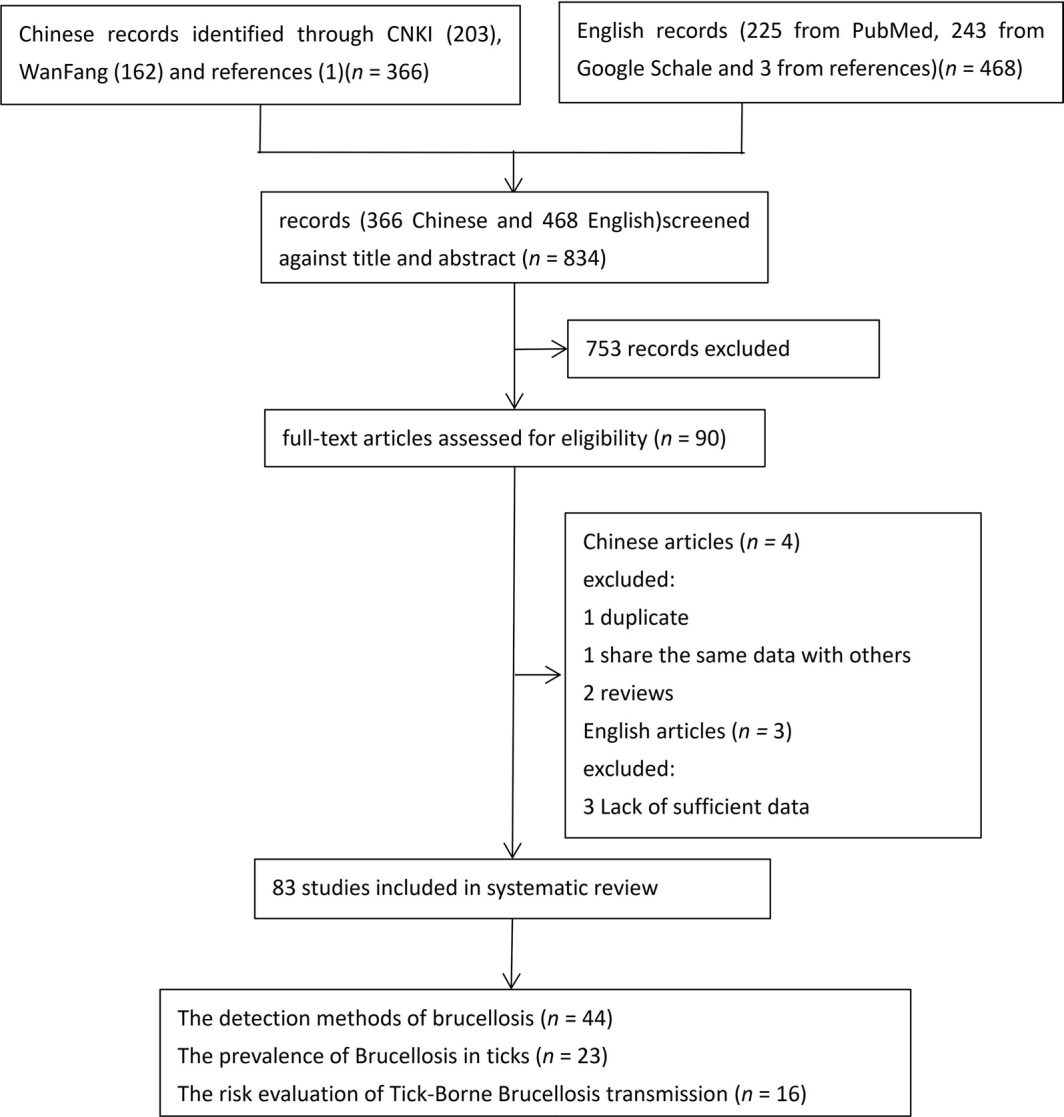
Flow diagram of literature search and study selection

The present study employed a rigorous set of inclusion and exclusion criteria to ensure the quality and relevance of the literature reviewed. As depicted in Fig. 2, a systematic search of various online databases was conducted to identify relevant references. Studies were considered eligible when they met the following eligibility criteria: (i) keywords related to *Brucella* detection; (ii) prevalence of brucellosis in ticks; (iii) experiments on brucellosis infection by ticks. After the articles were identified, they underwent a rigorous primary screening process. This step involved carefully reviewing the titles and abstracts of each article to determine its relevance to our study objectives. During the full-text screening, any articles that were found to be duplications of previously included studies were excluded. Furthermore, any articles that had significant overlap in terms of data with other studies were also excluded. Additionally, review articles were excluded during the full-text screening process. Lastly, articles that lacked sufficient data to address the research question were also excluded. This process helped to streamline the selection of articles for further review and analysis, thereby enhancing the overall validity and reliability of the study.

### Data extraction and analysis

The detailed characteristics of each study were extracted using a pre-designed data-collection Excel form. Information was recorded as follows: study information (the first author, year of publication, location); methodology (experimental method, in detail); characteristics of ticks (species, developmental stage, feeding status); sample size and reported prevalence of brucellosis in ticks. The prevalence data on brucellosis in ticks from all studies were collated, where possible.

### Quality assessment of included literature

We assessed the quality of the included articles with reference to Phyllis Munyiva Isaiah et al., using the Joanna Briggs Institute Prevalence Critical Appraisal Tool. All selected studies were scored using the 10 quality control items suggested by the tool. A score of one was awarded for each item fulfilled while a zero score was awarded for each unmet item. Score aggregates were generated and studies were classified as either low (0–3), moderate (4–6), or high (7–10) quality (see Additional file 2).

### Gap analysis

After extensive research, identified gaps were categorized into four distinct types: insufficient or imprecise information, biased information, inconsistency or unknown consistency, and a lack of pertinent information. To address these gaps in an effective manner, we assigned priority ratings to each item. This pragmatic and objective approach served as a valuable guide for future improvement strategies, allowing for a more focused and efficient implementation.

## Results

### Selection of evidence

As depicted in the illustration provided in Fig. 2, we thoroughly searched electronic databases, which yielded 843 papers. After carefully screening each document based on title and abstract relevance, 753 articles were excluded, while 90 articles met the criteria for full-text screening. Eventually, 83 articles were deemed eligible for inclusion in our research. Among the 83 studies analyzed, a total of 23 studies focused on prevalence, 44 examined detection methods, and 16 evaluated risk factors associated with the tick-borne brucellosis.

### Methods for detection of brucellosis in ticks

Ticks are known to be potential vectors for transmitting brucellosis. Detecting brucellosis in ticks is crucial for understanding the epidemiology of the disease and implementing appropriate control measures. The present study evaluated the current diagnostic methods for brucellosis, including pathogenic, serological, and molecular biology techniques in Table 1. We also summarize the currently available methods for the detection of brucellosis in ticks.

**Table 1:** Commonly used methods for *Brucella* detection

Method		Sen (%)/Spe (%)	Pros/Cons
Culture^a^	Lysis centrifugation^a^	Sen: NA Spe: 70.00–90.00% [19]	Pros: species-level identification and genotyping, antibiotic resistance detection; Cons: slow growth, laboratory safety issues, etc. [20]
	Bone marrow culture	Sen: NA Spe: 50.00–97.00% [19]	
	Ruiz-Castañeda method	Sen: NA Spe: 30.00–80.00% [19]	
Serology	RBT	Sen: 92.00–98.90% Spe: > 99.00% [21]	Pros: diagnosis is based on the test results of two or more methods; simple and time-saving, large-scale testing [22]; Cons: species-specific identification is not rapid enough and requires immunological identification of infected animals [23]
	MRT	Sen: 80.00–86.60% Spe: 100.00% [24]	
	ELISA	Sen: 96.60–100.00% Spe: 100.00% [21]	
	FPA	Sen: 97.90% Spe: 96.10% [20]	
	CFT	Sen: NA Spe: NA	
	SAT	Sen: 80.40%–99.50% Spe: 97.90%–99.00% [25]	
Conventional PCR^a^	*16S rRNA*^a^	Sen: 72.10% Spe: 100.00% [26]	Pros: fast, sensitive, accurate, and has a high safety factor; Cons: some factors inhibit DNA amplification, showing low sensitivity and thus false negative [27]
	*Bscp31*^a^	Sen: 92.72–98.30% Spe: 100.00% [28]	
	*IS711*^a^	Sen: 100.00% Spe: 100.00% [28]	
	*Omp22*^a^	Sen: NA Spe: NA	
	*Omp2*	Sen: 61.81% Spe: 100.00% [29]	
Multiplex-PCR	*Omp31*, *Omp25b*, *WboA*, *RpsL*, *Bp26,* etc	Sen: 85.38–94.11% Spe: 98.06–98.76% [30]	Pros: time-saving and labor-saving, suitable for large-scale detection and identification of *Brucella* species [31]; Cons: possible non-specific expansion, false positives
Real-time PCR	*Omp31*	Sen: 98.00% Spe: 100.00% [32]	Pros: high specificity, no need for gel electrophoresis, and can avoid contamination Cons: prone to form aerosol, non-specific expansion, false positives [33]
	*Bscp31*	Sen: 91.90% Spe: 95.40% [34]	
	*Acetyl-CoA*	Sen: NA Spe: 100.00% [35]	
Western blotting	*Omp28*	Sen: 93.00–97.00% Spe: 98.00–99.00% [36]	Pros: facilitate the distinction between brucellosis and other infections caused by cross-reactive bacteria [37]; Cons: antibody production may be more affected by individual strains than bacterial species, so immunodominant protein expression may vary between in vitro and in vivo culture conditions [38]
mNGS^a^	NA	Sen: 100.00% Spe: 90.00%	Pros: detection of rare, novel, and co-infected pathogens and also with advantages in resistance detection [39]; Cons: high cost, complex testing and interpretation, and slow turnaround time [40]
LAMP	*Omp25*	Sen: 100.00% Spe: 97.80% [32]	Pros: the reaction time is short, and the results are visualized for rapid detection [41] Cons: difficult primer design; prone to non-specific amplification or false positive [42]
RPA	*Bscp31*	Sen: NA Spe: 94.00% [43]	Pros: simple operation, fast response, low requirements on equipment [44] Cons: complicated and expensive and false positives
	*Bp26*	Sen: 97.00% Spe: 94.90% [2]	
	*Omp31*	Sen: NA Spe: NA [45]	

Sen: Sensitivity; Spe: Specificity; NA: Not available; RBT: Rose Bengal Test; MRT: Milk Ring Test; ELISA: Enzyme-linked immunosorbent assay; FPA: Fluorescence polarisation assay; CFT: Complement fixation test; SAT: Standard tube agglutination test; LAMP: Loop-mediated isothermal amplification; RPA: Recombinase polymerase amplification; mNGS: Metagenomic next-generation sequencing

^a^*Brucella* detection methods have been reported in ticks

One of the most widely employed techniques for identifying *Brucella* in ticks is through culture-based methods [19]. This entails the isolation of the bacteria from tick samples, followed by cultivation in specialized culture media. The ticks are subjected to surface sterilization prior to the aseptic removal of their internal organs or tissues for culture. The cultured samples are then incubated under precise conditions to encourage bacterial growth. Upon the emergence of colonies, they can be subjected to additional identification processes utilizing either biochemical tests or molecular techniques.

Molecular techniques have revolutionized the detection of various pathogens, including brucellosis in ticks [46]. Polymerase chain reaction (PCR) has emerged as a highly effective molecular method for amplifying specific DNA sequences of *Brucella* bacteria present in tick samples. PCR is capable of detecting even low levels of bacterial DNA, providing an unparalleled level of sensitivity and specificity. Several PCR-based assays, including conventional PCR, real-time PCR, nested PCR, and multiplex PCR [33, 47], have been developed to facilitate rapid and accurate detection of *Brucella* in tick samples based on different molecular markers (16S rRNA, *Bscp31*, *IS711*, *Omp22*) [48]. *Bscp31* and *Omp22* have been highlighted as reliable and frequently used markers for brucellosis detection in ticks. These cutting-edge molecular techniques offer a promising alternative to conventional, culture-based methods and have opened up new avenues for tick-borne *Brucella* detection and research.

Recently, rapid *Brucella* detection techniques such as loop-mediated isothermal amplification (LAMP) and recombinase polymerase amplification (RPA) have been developed [49, 50]. These techniques could operate under constant temperature conditions, do not require expensive equipment, and provide rapid results, demonstrating the great potential in improving the diagnosis of brucellosis. Furthermore, the advent of metagenomic next-generation sequencing (mNGS) technologies has scaled up the possibilities for detecting and characterizing microbial pathogens. mNGS enables the simultaneous sequencing of millions of DNA fragments, providing a comprehensive view of the microbial community present in tick samples. Through the analysis of the sequence data, it is possible to identify the presence of *Brucella* DNA in ticks and determine its genetic characteristics. Notably, mNGS provides a more detailed view of the microbial composition and functional potential of tick-associated bacteria, including *Brucella* [51]. Overall, several methods can currently be employed to detect brucellosis in ticks, providing valuable insights into the epidemiology and transmission dynamics of this zoonotic disease. However, there is no consensus on which approach is more sensitive to detecting brucellosis in ticks. Further research and comparative studies are required to determine the sensitivity of different molecular markers for brucellosis detection in ticks.

### Presence and prevalence of brucellosis in ticks

Several studies have shown that ticks can carry *Brucella*. According to the earliest survey conducted in 1937, ticks were found to be capable of carrying *Brucella* for an extended period during experimental conditions. Additionally, the live bacteria were identified in the feces of the ticks [52]. Since the initial study, there have been ongoing studies on the detection of *Brucella* in different tick species. Through conducting a thorough review of literature on the presence or prevalence of brucellosis in ticks, we revealed that brucellosis could be present among 16 different tick species, and the overall prevalence of brucellosis in ticks was about 33.87% (2524/7452) ranging from 0.00% to 87.80%, as highlighted in Table 2.

**Table 2:** The presence and prevalence of brucellosis in ticks from literature references

Tick species	Developmental stage	Feeding status	*Brucella* strains	Prevalence	Methods
*Hyalomma marginatum* *Hyalomma savignyi*	Adult	Parasitic	*Brucella*	NA	Bacteria isolation [53]
*Dermacentor*	Larval nymph	Parasitic	*B. melitensi*	NA	Animal experiment [54]
*Annulatus*	Adult	Parasitic	*Brucella*	60.00% (6/10)	Bacteria isolation [55]
*Boophilus*				80.00% (8/10)	
*Hyalomma marginatum* *Dermacentor nuttalli*	Adult	Parasitic	*B. abortus*	NA	Animal experiment [56]
*Dermacentor marginatus* *Dermacentor albipictus*	Adult	Parasitic	*B. melitensi*	NA	Animal experiment [57]
*Dermacentor nuttalli* *Dermacentor sinicus*	Egg Adult	Parasitic	*Brucella*	NA	Animal experiment [58]
*Dermacentor daghestanicu*	Adult	Parasitic	*Brucella*	NA	Animal experiment [59]
*Rhipicephalus sanguineus*	Adult	Parasitic	*B. canis*	NA	Bacteria isolation [60]
*Hyalomma anatolicum*	Adult	Parasitic	*B. abortus*	45.00% (225/500)	Bacteria isolation [61]
*Rhipicephalus microplus*				70.00% (350/500)	
*Dermacentor nuttalli*	Adult	Parasitic	*B. melitensi*	53.38% (1020/1911)^a^	PCR [62]
*Boophilus*	Adult	Parasitic	*B. abortus*	NA	PCR [48]
*Dermacentor marginatus*	Egg	Parasitic	*Brucella*	4.60% (16/350)	PCR [63]
	Larval			40.90% (90/220)	
	Adult		*B. melitensi*	4.13% (10/242)	
			*B. abortus*	5.56% (6/108)	
*Dermacentor nuttalli*	Adult	Parasitic	*Brucella*	27.54% (320/1162)	PCR [64]
*Dermacentor nuttalli*	Adult	Parasitic	*Brucella*	18.81% (60/319)	PCR [65]
*Dermacentor marginatus*				41.90% (75/179)	
				34.26% (37/108)	
*Rhipicephalus turanicus*				11.59% (8/69)	
*Haemaphysalis punctata*				12.50% (9/72)	
*Hyalomma asiaticum*					
		Non-parasitic		7.42% (25/337)	
*Hyalomma anatolicum*	Adult	Parasitic	*Brucella*	44.40% (111/250)	PCR [66]
*Dermacentor nuttalli*				14.40% (36/250)	
*Dermacentor marginatus*				20.00% (50/250)	
*Haemaphysalis longicornis*	Adult	Parasitic	*Brucella*	24.26% (41/169)	PCR [67]
*Rhipicephalus turanicus*	Adult	Parasitic	*B. canis*	4.82% (21/436)	PCR [68]
*Ixodes ricinus*	Adult	Non-parasitic	*Brucella*	NA	mNGS [69]
*Dermacentor silvarum*	Adult	Parasitic	*Brucella*	NA	mNGS [70]

NA: Not available; mNGS: metagenomic next-generation sequencing

^a^Average prevalence from different population, range: 0.00–87.80% (36/41)

In addition, *Brucella* has been observed at various developmental stages of ticks. For example, Gudoshnik and Wang et al. [57, 63] found that *Brucella* could be present in different developmental stages of *D. marginatus* by animal experiments. Moreover, the prevalence rate of the *Brucella* was 40.9% in larvae 4.6% in female ticks, which developed from the same batch of *Brucella*-positive eggs. Researchers from Mexico, China, and other countries have confirmed that *Brucella* can be transmitted via vertical route [55, 56, 58].

**Fig. 3:**
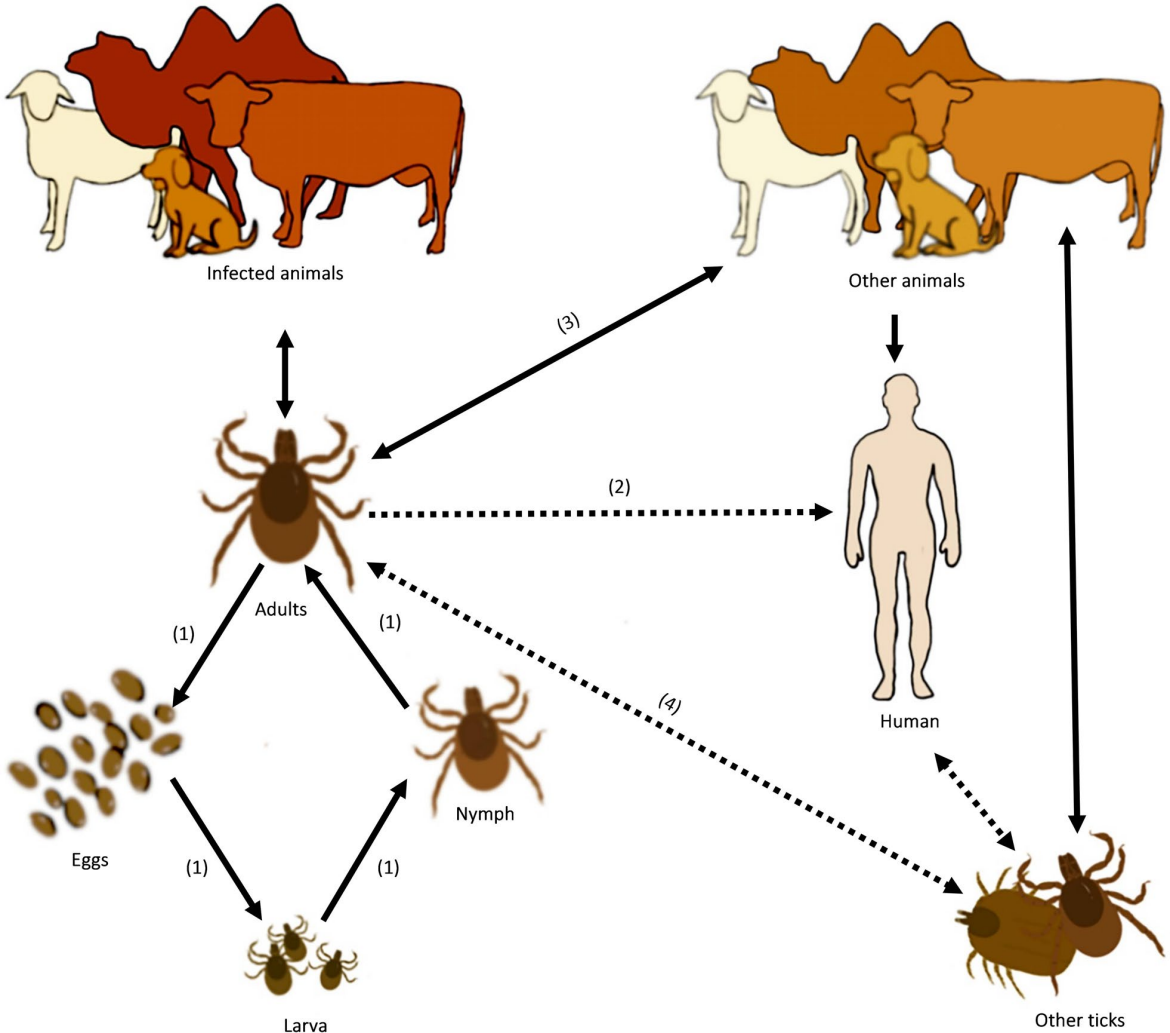
Risk evaluation of tick-borne brucellosis transmission. (**1)** After adult ticks become infected with *Brucella*, there is the potential for vertical transmission to occur, leading to the infection of different developmental stages of ticks through eggs. (**2)** Infected ticks have the capability to transmit *Brucella* to humans. (**3)** Ticks may also transmit *Brucella* after biting healthy animals. (**4)** There is a possibility of “co-feeding transmission” where infected, healthy ticks feed on the same host. This complex mode of transmission highlights the intricate interactions between ticks, hosts, and pathogens in natural ecosystems. Note: the solid lines on the diagram denote confirmed transmission routes, while the dotted lines indicate potential transmission routes

Moreover, recent research has revealed that the presence of *Brucella* can be identified in various tissues and organs of ticks (Fig. 3). According to Huang et al. [62], they were able to use a fluorescent quantitative PCR technique to detect the copy number of the *Bcsp31* gene of *Brucella* in the salivary gland and midgut of *D. nuttalli*. The study was successful in detecting the BCSP31 protein of *Brucella* at the protein level as well. Furthermore, the ability of *Brucella* to adapt to the intracellular environment of ticks' primary cells has been demonstrated in a study where primary cells from the salivary glands and midgut tissue of *D. nuttalli* were isolated and cultured in vitro. This finding suggests that *Brucella* has the capability to survive and replicate within tick cells, which may play a role in its transmission and persistence within tick populations [71].

### Risk assessment of tick-borne brucellosis transmission

A series of experiments were conducted to investigate the potential role of ticks in the transmission of *Brucella* and their ability to infect healthy animals. One set of experiments involved engorged female ticks obtained from guinea pigs infected with *Brucella* [56, 57]. These ticks were cultured, and a homogenate was injected into healthy guinea pigs. The results showed positive test results for *Brucella* in the injected guinea pigs, indicating that transmission may had occurred. Another similar experiment involved ticks obtained from sheep infected with brucellosis. These ticks were placed on healthy guinea pigs, allowing them to feed and potentially transmit the bacteria. After the ticks were fed, detection was conducted on the guinea pigs to detect any signs of infection. The serological tests were positive, further supporting the potential ability of ticks in transmitting *Brucella* [53, 54]. These experiments shed light on the potential role of ticks as vectors for transmitting *Brucella*. The ability of ticks to communicate *Brucella* has been experimentally demonstrated in some studies. For example, a study published in 1979 showed that the soft tick (*Ornithodoros moubata*) could acquire and transmit *B. abortus*, the causative agent of bovine brucellosis [72]. Several studies have investigated the role of ticks in the transmission of *Brucella* species. One study in Spain found that ticks collected from livestock were positive for *Brucella* DNA, suggesting their potential role as vectors for brucellosis transmission [73]. Similarly, another study conducted in Iran detected *Brucella* DNA in ticks collected from sheep and goats [74]. While further research is needed to fully understand the mechanisms and significance of tick-borne transmission in brucellosis, these findings highlight the importance of considering ticks as potential sources of infection.

Moreover, multivariable analyses have been conducted to investigate the factors contributing to the risk of brucellosis on farms. These analyses have shown that improvements in farm biosecurity and hygiene practices can significantly reduce the risk of brucellosis. Additionally, some studies suggested that tick bites compromise the immune response of infected animals, which may lead to increased susceptibility of cattle to *Brucella* infection [75]. It is important to note that while ticks may play a role in the transmission of brucellosis, they are not the sole means of transmission.

The transmission of brucellosis to humans by ticks has been discussed scarcely in the literature. Upon reviewing the database, we found only three case reports from 1963 to 2019 regarding brucellosis transmission to humans. One of the cases involved a slaughterhouse worker in the United Kingdom who contracted brucellosis after being bitten by a tick while at work. Initially, it was thought that the worker contracted brucellosis from the slaughtered animal, but a tick was removed from under the worker's shoulder before the onset of the illness [76]. The investigators recommended that attention be given to the possibility of vector brucellosis transmission. In 2011, a study by Simsek and colleagues in Turkey reported the cases of six patients with brucellosis, one of whom had a possible brucellosis infection caused by a tick bite [77]. A decade later, in 2019, researchers identified that one patient had not come into contact with unpasteurized dairy products, and instead, the brucellosis was linked to repeated tick bites [78]. The identification and confirmation of tick-borne brucellosis cases pose a significant challenge due to the diverse range of potential sources for the pathogen, including unpasteurized dairy and meat products [79]. The complexity of establishing epidemiological links necessitates rigorous biosafety protocols and ethical considerations during experimental validation. These limitations make it difficult for researchers to conduct comprehensive investigations into the disease, despite the severe implications for animal and human health [80, 81]. Further research is required to address these challenges and advance our understanding of this elusive pathogen.

It has been traditionally believed that arthropods, specifically ticks, only transmit pathogenic microorganisms by biting their hosts or through vertical propagation. However, recent research has suggested that this view may be flawed. It has been observed that ticks living and feeding in close spatiotemporal proximity can also lead to the transmission of pathogenic microorganisms between ticks, termed co-feeding transmission [82]. This presents new opportunities and avenues for transmission. Although co-feeding transmission has only been documented in some pathogen infection, such as TBE group flaviviruses [83], and *Borrelia burgdorferi* [84], its possible mechanism in tick-borne *Brucella* warrants further investigation.

### Gap analysis for the role of ticks in the transmission of brucellosis

By undertaking a detailed scoping review, we found that the literature on tick-borne *Brucella* transmission to animals or humans was scarce, with several factors contributing to the gap in the role of ticks in the transmission of brucellosis. First, research on TBDs is presently limited due to ethical and biosafety factors that prevent *Brucella* research from being carried out in ordinary laboratories. Second, there is a lack of solid evidence supporting ticks as effective vectors, with insufficient data from animal experiments, clinical trials, and large-scale epidemiological studies. Third, current detection methods for *Brucella* in ticks lack updates, and more convenient tools are needed. Finally, the intricate process by which ticks transmit *Brucella* bacteria, as well as the variations in vector capacity among different tick species, remain largely unresolved and require further investigation. This critical analysis seeks to better understand the reasons for this gap and outlines strategies to bridge it. Hopefully, this will provide valuable information for the biological field on how to not simply bridge but also close this gap, thereby creating more substantial evidence for tick-borne *Brucella*.

## Discussion

The increasing prevalence of zoonotic diseases globally has been a source of mounting apprehension. Recent findings have revealed that the majority of zoonotic diseases, approximately 71.80%, originate from wild animals (22.80% are vector-borne diseases) [85]. Ticks have been identified as the primary culprits for transmitting a vast array of diseases to domestic animals compared to other arthropods [16, 86]. Despite many studies on the relationship between brucellosis transmission and ticks, ticks' precise role in transmitting this disease and the associated risks remain poorly understood.

More than 800 species of ticks in 18 genera have been identified in the world, of which about 80 species of ticks are considered to be the vectors of disease transmission in the world. Understanding ticks’ biology, its ecological roles, and vectors for numerous pathogens is crucial for developing effective strategies to prevent and control tick-borne diseases and ensure the health of both humans and animals [87]. This scoping review indicated that *Brucella* had been detected in 16 species of ticks, although there remain tick species in which *Brucella* has yet to be identified. It must be noted that there is a lack of evidence to suggest that other tick species carry *Brucella* bacteria. This could be attributed to the rarity of brucellosis in the area. Regrettably, there is an evident dearth of research and testing in this regard, rendering it challenging to make definitive conclusions on the presence and prevalence of brucellosis in ticks.

As we explained above ticks can acquire *Brucella* bacteria by feeding on infected animals and subsequently transmit the bacteria to other hosts during subsequent blood meals. In addition to ticks, other blood-feeding parasites such as lice and fleas have also been implicated as potential vectors for brucellosis. A study conducted in Ethiopia found that lice collected from cattle were positive for *Brucella* DNA, suggesting their potential role in transmitting the bacteria [88]. Similarly, a study conducted in Mexico detected *Brucella* DNA in fleas collected from dogs [55]. It is important to note that while these studies provide evidence for the potential role of ticks and other blood-feeding parasites as vectors for brucellosis, further research is needed to understand the extent of their involvement in disease transmission fully.

As mentioned above, the advancement of *Brucella* detection methods has been of paramount importance in studying the relationship between ticks and *Brucella*. The emergence of PCR technology has revolutionized the detection of *Brucella* in ticks, making it more secure, rapid, and accessible on a large scale. In addition, the development of metagenome sequencing technology has enhanced the probability of detecting brucellosis in ticks. However, it is important to explore and analyze whether the newly discovered and improved technical detection methods for brucellosis can be effectively applied to ticks.

There is little literature on tick-borne brucellosis in humans, and empirical evidence is limited mainly due to various factors such as biosecurity measures and the numerous routes of human brucellosis infection. Humans may infect *Brucella* through multiple ways, including consuming contaminated raw milk and dairy products, close contact with infected animals, or laboratory exposure [89]. The transmission route of brucellosis can be challenging to determine when patients report both a history of tick bites and contact with infected animals and their products, making it difficult to ascertain whether ticks are an effective vector in transmission. Additional research is needed better to understand the mechanisms of human infection by ticks and to bridge this imperative knowledge gap.

To effectively conduct a risk assessment for tick-borne brucellosis transmission, several factors need to be considered, including the prevalence of *Brucella*-infected ticks in the area, the behavior and habitat of the ticks, and the potential for human exposure. The prevalence of brucellosis in ticks can be obtained through surveillance programs conducted by public health agencies or research institutions. Tick surveillance data can help identify areas with a higher risk of tick-borne brucellosis transmission. Identifying high-risk areas based on tick behavior and habitat can help guide preventive measures. Certain geographical regions may have a higher prevalence of infected ticks, increasing the risk of transmission. Human exposure, such as outdoor activities, occupation, and geographical location, play a role in determining the likelihood of encountering infected ticks. Individuals who spend a significant amount of time outdoors, such as hikers, campers, or outdoor workers, are at a higher risk of tick bites.

Given the current state of environmental changes, closely monitoring the potential impact on tick distribution and the associated risk of tick-borne brucellosis is of utmost importance. First of all, ticks are highly sensitive to environmental changes, as they spend most of their life cycle in this setting. Their survival is dependent on several climatic variables, including vegetation and the presence of appropriate hosts. Studies have found that climatic changes, such as rising temperatures, can positively affect ticks' survival. This can lead to an increase in number, activity, range of ticks and the potential for these parasites to spread, become established, and persist in new locations [90, 91]. Second, climate change affects both the reproduction hosts and the reservoir hosts involved in the tick lifecycle and spread of TBDs, respectively. Increasing temperatures will expand the distribution range of hosts as well as their abundance and activity. In addition, as a result of climate change, people may resume outdoor activity earlier in the spring and maintain it longer in the fall. With the increase in length of exposure to tick habitat, combined with an extended season of tick activity, the likelihood of encountering ticks carrying unknown pathogens increases, thereby elevating the risk of human infection with brucellosis and other tick-borne illnesses [92]. This fits with another concept, the emergence and spread of zoonosis are heavily influenced by a variety of factors, many of which are related to climate change and environmental factors. Habitat variation and atmospheric and climatic changes are among the most significant of these factors, and they can significantly impact the spread of diseases from animals to humans [93]. Therefore, in the context of adaptation to climate change, we must remain vigilant in our efforts to mitigate these risks and safeguard against potential health hazards posed by ticks.

## Conclusions

The present scoping review provides a comprehensive overview of the existing literature about the role of ticks in brucellosis transmission. We have revealed that *Brucella* is present across a variety of developmental stages in ticks, indicating the potential for widespread transmission and dissemination of this pathogen within and among tick populations. Available detection methods for brucellosis detection were also presented and evaluated. The potential role of ticks as vectors of brucellosis and the risks they may pose suggests the need for further studies. The assessment of the role and specific mechanisms of ticks in the epidemiology and transmission of brucellosis revealed the need for other in-depth studies, as well as the availability of quick and safe research methods to explore *Brucella* in ticks. The increased risk of tick-borne diseases is highlighted as essential to better understanding the interactions between ticks, *Brucella*, animal hosts, humans, and the environment.

### Supplementary Information

**Additional file 1.** Databases and search strategies.

**Additional file 2.** Quality assessment of included literatures.

## Data Availability

Not applicable.

## Abbreviations

TBD: Tick-borne disease
RBT: Rose Bengal Test
MRT: Milk Ring Test
ELISA: Enzyme-linked immunosorbent assay
FPA: Fluorescence polarisation assay
CFT: Complement fixation test
SAT: Standard tube agglutination test
LAMP: Loop-mediated isothermal amplification
RPA: Recombinase polymerase amplification
mNGS: Metagenomic next-generation sequencing
PCR: Polymerase chain reaction
