# Supplementary material for: Evidence-practice gap analysis in the role of tick in brucellosis transmission: a scoping review
Source: Infect Dis Poverty. 2024 Jan 8;13:3. doi: 10.1186/s40249-023-01170-4 (PMC10773131; doi:10.1186/s40249-023-01170-4)
Supplement: Supplementary file 1 — Additional file 1. Databases and search strategies. [file 40249_2023_1170_MOESM1_ESM.docx]

**Additional file 1:** Databases and search strategies

**Table S1: Search terms used in Chinese bibliographic databases**

| **Search cluster** | **Search terms (China National Knowledge Infrastructure and Wanfang)** |
| --- | --- |
| tick | (tick* OR blood-sucking arthropod) AND |
| *Brucella* | (*Brucella** OR brucellosis OR *B.abortus* OR B.ovis OR *B.melitensi* ) AND |
| the prevalence of brucellosis in ticks | ((“brucellosis in ticks” OR “tick-borne brucellosis* ” OR *Brucella*).ti,ab,kw) AND |
| the detection methods of brucellosis | ((“methods for detecting brucellosis” OR “brucellosis diagnosis*” OR diagnosis OR diagnosing OR test or testing).ti,ab,kw) AND |
| the risk of tick-borne brucellosis | ( “tick-borne disease risk” OR “tick-borne brucellosis” OR *Brucella*).ti,ab,kw) AND |

**Table S2: Search terms used in English bibliographic databases**

| **Search cluster** | **Search terms (Google Scholar and PubMed)** |
| --- | --- |
| tick | (tick* OR blood-sucking arthropod) AND |
| *Brucella* | (*Brucell*a* OR brucellosis) AND |
| the prevalence of brucellosis in ticks | ((“brucellosis in ticks” OR “tick-borne brucellosis* ” OR *Brucella*).ti,ab,kw) AND |
| the detection methods of brucellosis | ((“methods for detecting brucellosis” OR “brucellosis diagnosis*” OR diagnosis OR diagnosing OR test or testing).ti,ab,kw) AND |
| the risk of tick-borne brucellosis | ( “tick-borne disease risk” OR “tick-borne brucellosis” OR *Brucella*).ti,ab,kw) AND |
